# Supplementary material for: Prevalence of Sarcopenia and Its Impact on Cardiovascular Events and Mortality among Dialysis Patients: A Systematic Review and Meta-Analysis
Source: Nutrients. 2022 Sep 30;14(19):4077. doi: 10.3390/nu14194077 (PMC9572026; doi:10.3390/nu14194077)
Supplement: Supplementary file 1 [file nutrients-14-04077-s001.zip › nutrients-1899156-supplementary.pdf]

Supplementary Material

Table S1. Search strategy in each database.

PubMed

(("Sarcopenia"[Mesh] OR Sarcopenia*[tiab])) AND ("Kidney Failure, Chronic"[Mesh] OR End-Stage Kidney Disease [tiab] OR Disease, End-Stage Kidney [tiab] OR End Stage Kidney Disease [tiab] OR Kidney Disease, End-Stage [tiab] OR Chronic Kidney Failure [tiab] OR End-Stage Renal Disease [tiab] OR Disease, End-Stage Renal [tiab] OR End Stage Renal Disease [tiab] OR Renal Disease, End-Stage [tiab] OR Renal Disease, End Stage [tiab] OR Renal Failure, End-Stage [tiab] OR End-Stage Renal Failure [tiab] OR Renal Failure, End Stage [tiab] OR Renal Failure, Chronic [tiab] OR Chronic Renal Failure [tiab] OR ESRD [tiab] OR ESKD [tiab] OR dialy* [tiab] OR hemodialy* [tiab])

Scopus

TITLE-ABS-KEY (sarcopeni* AND (end-stage AND kidney AND disease OR disease, AND end-stage AND kidney OR end AND stage AND kidney AND disease OR kidney AND disease, AND end-stage OR chronic AND kidney AND failure OR end-stage AND renal AND disease OR disease, AND end-stage AND renal OR end AND stage AND renal AND disease OR renal AND disease, AND end-stage OR renal AND disease, AND end AND stage OR renal AND failure, AND end-stage OR end-stage AND renal AND failure OR renal AND failure, AND end AND stage OR renal AND failure, AND chronic OR chronic AND renal AND failure OR esrd OR eskd OR dialy* OR hemodialy*))

Cochrane Library

sarcopeni* AND (end-stage AND kidney AND disease OR disease, AND end-stage AND kidney OR end AND stage AND kidney AND disease OR kidney AND disease, AND end-stage OR chronic AND kidney AND failure OR end-stage AND renal AND disease OR disease, AND end-stage AND renal OR end AND stage AND renal AND disease OR renal AND disease, AND end-stage OR renal AND disease, AND end AND stage OR renal AND failure, AND end-stage OR end-stage AND renal AND failure OR renal AND failure, AND end AND stage OR renal AND failure, AND chronic OR chronic AND renal AND failure OR esrd OR eskd OR dialy* OR hemodialy*))

Table S2. Risk of bias assessment using the Newcastle-Ottawa Scale adapted for cross-sectional studies.[1, 2].

Cross-sectional studies								
	Selection				Comparability	Outcome		Quality
First author (year)	Q1	Q2	Q3	Q4	Q1	Q1	Q2	
Lamarca F. (2014)[3]	*		*	**		**	*	7 (Good)
Hotta C. (2015)[4]			*	**		**	*	6 (Satisfactory)
Bataille S. (2016)[5]	*		*	**		**	*	7 (Good)
Ren H. (2016)[6]	*		*	**		**	*	7 (Good)
As'habi A. (2018)[7]	*		*	**		**	*	7 (Good)
Abro A. (2018)[8]	*		*	**		**	*	7 (Good)
Yoowannakul S. (2018)[9]	*		*	**		**	*	7 (Good)
Lin Y. (2018)[10]	*		*	**		**	*	7 (Good)
Slee A. (2020)[11]	*		*	**		**	*	7 (Good)
Wang M. (2021)[12]	*		*	**		**	*	7 (Good)
Hortegal EVF. (2020)[13]	*		*	**		**	*	7 (Good)
Oliveira E. (2020)[14]	*		*	**		**	*	7 (Good)
Lee H. (2020)[15]	*		*	**		**	*	7 (Good)
Medeiros M. (2020)[16]	*		*	**		**	*	7 (Good)
Umakanthan J. (2021)[17]	*		*	**		**	*	7 (Good)
Do J. (2021)[18]	*		*	**		**	*	7 (Good)
Abdala R. (2021)[19]	*		*	**		**	*	7 (Good)
Yuenyongchaiwat K. (2021)[20]	*		*	**		**	*	7 (Good)
Cheng D. (2021)[21]	*		*	**		**	*	7 (Good)
Matsuzawa R (2021)[22]	*		*	**		**	*	7 (Good)
Rosa C. (2021)[23]	*		*	**		**	*	7 (Good)
Lin Y. (2022)[24]	*		*	**		**	*	7 (Good)
Yasar E. (2022)[25]	*		*	**		**	*	7 (Good)
Ding Y. (2022)[26]	*		*	**		**	*	7 (Good)
Kurajoh M. (2022)[27]	*		*	**		**	*	7 (Good)

Table S3. Risk of bias assessment using the Newcastle-Ottawa scale for cohort studies [1, 2].

Cohort Studies									
	Selection				Comparability	Outcome			Quality
First author (year)	Q1	Q2	Q3	Q4	Q1	Q1	Q2	Q3	
Isoyama N. (2014)[28]	*	*	*		**	*	*		Good
Kittiskulnam P. (2017)[29]	*	*	*		**	*	*		Good
Kamijo Y. (2018)[30]	*	*	*		**	*	*	*	Good
Giglio J. (2018)[31]	*	*	*		**	*	*		Good
Kim J. (2019)[32]	*	*	*		**	*	*		Good
Mori K. (2019)[33]	*	*	*		**	*	*	*	Good
Lin Y. (2020)[34]	*	*	*		**	*	*	*	Good
Chiang J. (2019)[35]	*	*	*		**	*	*		Good
Macedo C. (2021)[36]	*	*	*		**	*	*		Good
Souweine J. (2021)[37]	*	*	*		**	*	*	*	Good
Kim C. (2021)[38]	*	*	*		**	*	*	*	Good
Davenport A. (2022)[39]	*	*	*		**	*	*		Good
Ferreira M. (2022)[40]	*	*	*		**	*	*	*	Good
Ishimura E. (2022)[41]	*	*	*		**	*	*	*	Good
Sanchez-Tocino M. (2022)[42]	*	*	*		**	*	*	*	Good
Hayashi H. (2022)[43]	*	*	*		**	*	*	*	Good

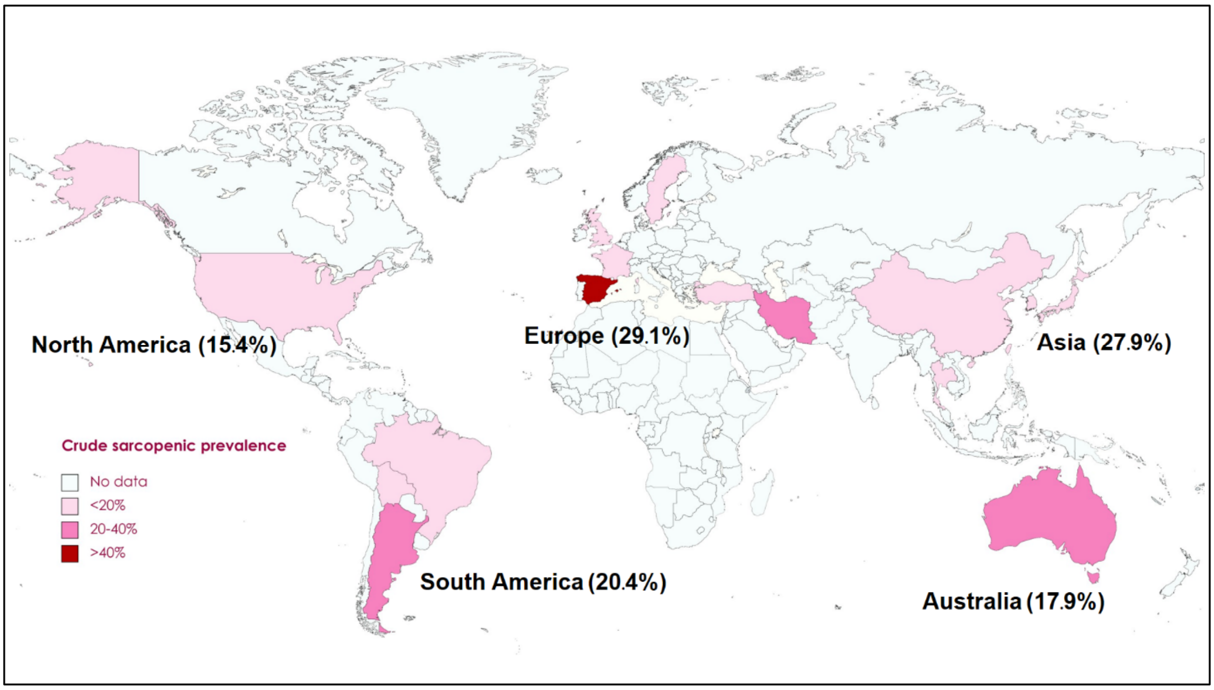

Figure S1. **Prevalence of sarcopenia among dialysis patients worldwide reported from studies published during 2014-2022**. **Color gradation reflects prevalence of sarcopenia in all included studies from each country** (**weighted averages within countries**).

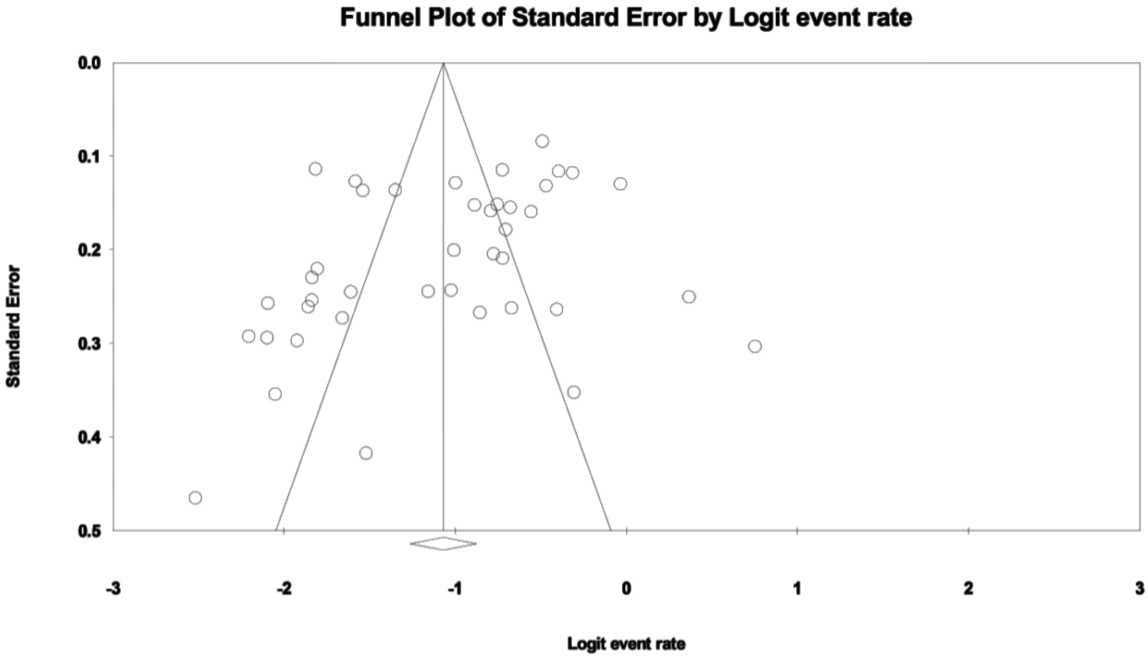

Figure S2. **Funnel plot showing publication bias in studies reporting the prevalence of sarcopenia in dialysis patients.**

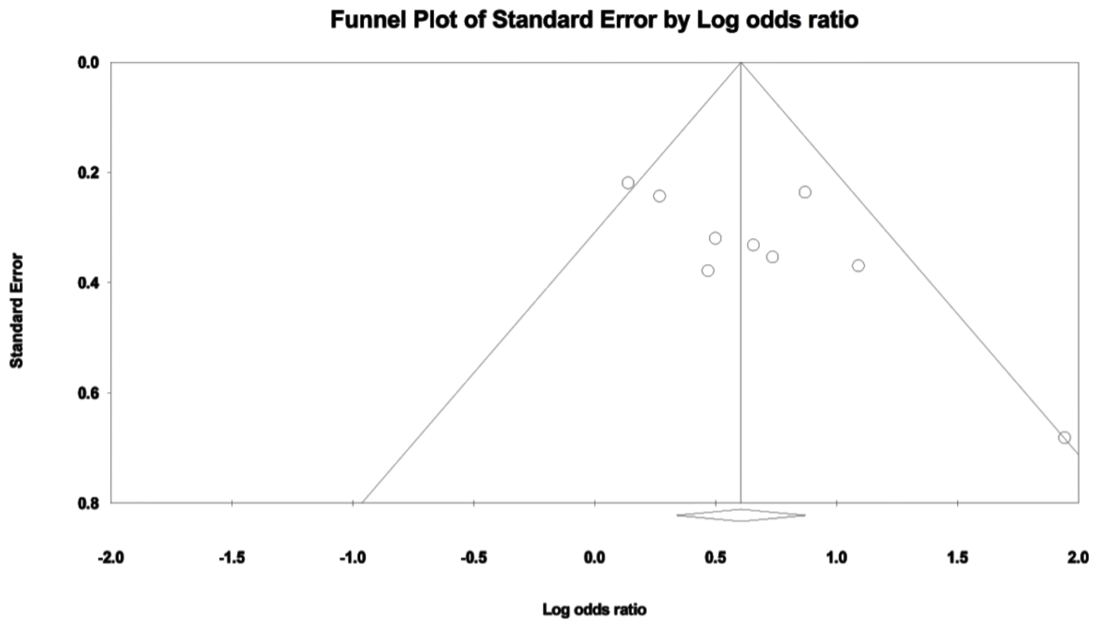

Figure S3. Funnel plot showing publication bias in studies reporting all-cause mortality of sarcopenia in dialysis patients.

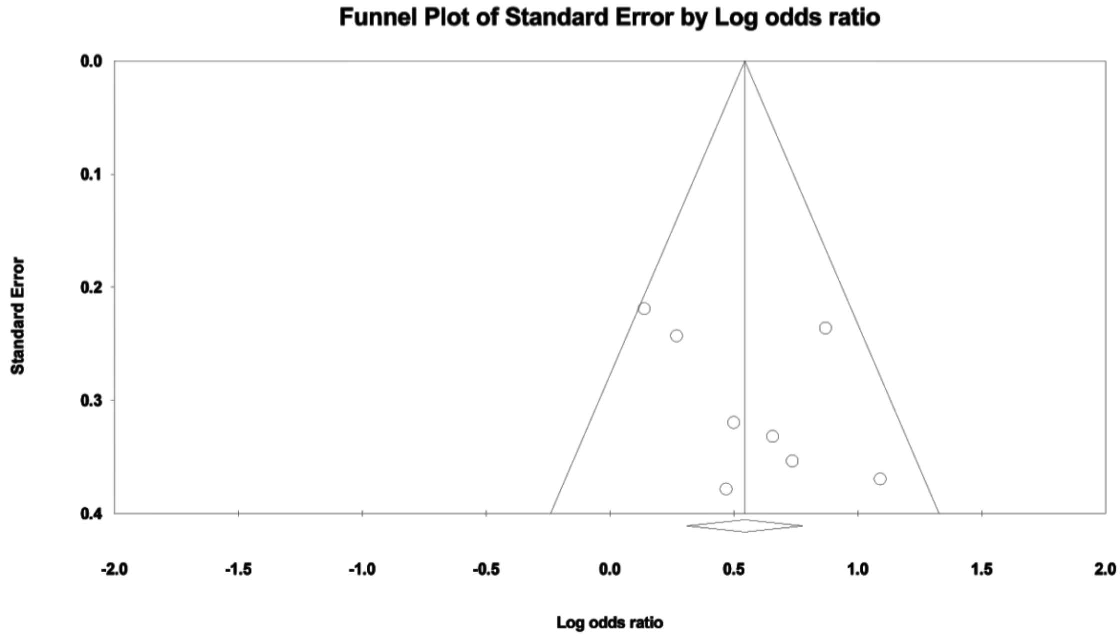

Figure S4. Funnel plot showing publication bias in studies reporting all-cause mortality of sarcopenia in dialysis patients after excluding Kim et al.

Reference

1. Wells, G.A.; Shea, B.; O'Connell, D.; Peterson, J.; Welch, V.; Losos, M.; Tugwell, P. The Newcastle-Ottawa Scale (NOS) for Assessing the Quality of Nonrandomised Studies in Meta-Analyses. Available online: http://www.ohri.ca/programs/clinical_epidemiology/oxford.asp (accessed on 9 April 2022).
2. Herzog, R.; Alvarez-Pasquin, M.J.; Diaz, C.; Del Barrio, J.L.; Estrada, J.M.; Gil, A. Are healthcare workers' intentions to vaccinate related to their knowledge, beliefs and attitudes? A systematic review. *BMC Public Health* **2013**, *13*, 154.
3. Lamarca, F.; Carrero, J.J.; Rodrigues, J.C.; Bigogno, F.G.; Fetter, R.L.; Avesani, C.M. Prevalence of sarcopenia in elderly maintenance hemodialysis patients: The impact of different diagnostic criteria. *J. Nutr. Health Aging* **2014**, *18*, 710–717.
4. Hotta, C.; Hiraki, K.; Wakamiya, A.; Otobe, Y.; Watanabe, S.; Izawa, K.P.; Kaneshiro, N.; Konno, Y.; Sakurada, T.; Shibagaki, Y.; et al. Relation of physical function and physical activity to sarcopenia in hemodialysis patients: A preliminary study. *Int. J. Cardiol.* **2015**, *191*, 198–200. https://doi.org/10.1016/j.ijcard.2015.05.005.
5. Bataille, S.; Serveaux, M.; Carreno, E.; Pedinielli, N.; Darmon, P.; Robert, A. The diagnosis of sarcopenia is mainly driven by muscle mass in hemodialysis patients. *Clin. Nutr.* **2017**, *36*, 1654–1660. https://doi.org/10.1016/j.clnu.2016.10.016.
6. Ren, H.; Gong, D.; Jia, F.; Xu, B.; Liu, Z. Sarcopenia in patients undergoing maintenance hemodialysis: Incidence rate, risk factors and its effect on survival risk. *Ren. Fail.* **2016**, *38*, 364–371. https://doi.org/10.3109/0886022x.2015.1132173.
7. As'Habi, A.; Najafi, I.; Tabibi, H.; Hedayati, M. Prevalence of Sarcopenia and Dynapenia and Their Determinants in Iranian Peritoneal Dialysis Patients. *Iran. J. Kidney Dis.* **2018**, *12*, 53–60.
8. Abro, A.; Delicata, L.-A.; Vongsanim, S.; Davenport, A. Differences in the prevalence of sarcopenia in peritoneal dialysis patients using hand grip strength and appendicular lean mass: Depends upon guideline definitions. *Eur. J. Clin. Nutr.* **2018**, *72*, 993–999. https://doi.org/10.1038/s41430-018-0238-3.
9. Yoowannakul, S.; Tangvoraphonkchai, K.; Vongsanim, S.; Mohamed, A.; Davenport, A. Differences in the prevalence of sarcopenia in haemodialysis patients: The effects of gender and ethnicity. *J. Hum. Nutr. Diet.* **2018**, *31*, 689–696. https://doi.org/10.1111/jhn.12555.
10. Lin, Y.-L.; Liou, H.-H.; Lai, Y.-H.; Wang, C.-H.; Kuo, C.-H.; Chen, S.-Y.; Hsu, B.-G. Decreased serum fatty acid binding protein 4 concentrations are associated with sarcopenia in chronic hemodialysis patients. *Clin. Chim. Acta* **2018**, *485*, 113–118. https://doi.org/10.1016/j.cca.2018.06.025.
11. Slee, A.; McKeaveney, C.; Adamson, G.; Davenport, A.; Farrington, K.; Fouque, D.; Kalantar-Zadeh, K.; Mallett, J.; Maxwell, A.P.; Mullan, R.; et al. Estimating the Prevalence of Muscle Wasting, Weakness, and Sarcopenia in Hemodialysis Patients. *J. Ren. Nutr.* **2020**, *30*, 313–321. https://doi.org/10.1053/j.jrn.2019.09.004.
12. Wang, M.; Liu, L.; Shen, X.; Li, Y.; He, Q. Assessing lean tissue by bioelectrical impedance analysis pre hemodialysis underestimates the prevalence of sarcopenia in maintenance hemodialysis patients. *Eur. J. Clin. Nutr.* **2021**, *75*, 1407–1413. https://doi.org/10.1038/s41430-020-00835-9.
13. Furtado, E.V.H.; Alves, J.J.D.A.; Santos, E.J.F.; Nunes, L.C.R.; Galvão, J.C.; Nunes, R.F.; Lula, D.D.A.; de Carvalho, S.C.R.; França, A.K.T.D.C.; dos Santos, E.M.; et al. Sarcopenia and inflammation in patients undergoing hemodialysis. *Nutr. Hosp.* **2020**, *37*, 855–862. https://doi.org/10.20960/nh.03068.
14. Oliveira, E.M.; Silva, R.P.D.; Lemos, M.; Burgos, M.; Costa, D.M.N.; Maio, R. Frequency of sarcopenia, cachexia, and associated factors in patients with chronic kidney disease in dialysis treatment. *Nutr. Hosp.* **2020**, *37*, 1157–1165.
15. Lee, H.; Kim, K.; Ahn, J.; Lee, D.R.; Lee, J.H.; Hwang, S.D. Association of nutritional status with osteoporosis, sarcopenia, and cognitive impairment in patients on hemodialysis. *Asia Pac. J. Clin. Nutr.* **2020**, *29*, 712–723.
16. Medeiros, M.C.; Rocha, N.; Bandeira, E.; Dantas, I.; Chaves, C.; Oliveira, M.; Bandeira, F. Serum Sclerostin, Body Composition, and Sarcopenia in Hemodialysis Patients with Diabetes. *Int. J. Nephrol.* **2020**, *2020*, 4596920. https://doi.org/10.1155/2020/4596920.
17. Umakanthan, M.; Li, J.W.; Sud, K.; Duque, G.; Guilfoyle, D.; Cho, K.; Brown, C.; Boersma, D.; Komala, M.G. Prevalence and Factors Associated with Sarcopenia in Patients on Maintenance Dialysis in Australia-A Single Centre, Cross-Sectional Study. *Nutrients* **2021**, *13*, 3284.
18. Do, J.Y.; Kim, A.Y.; Kang, S.H. Association Between Phase Angle and Sarcopenia in Patients Undergoing Peritoneal Dialysis. *Front. Nutr.* **2021**, *8*, 742081. https://doi.org/10.3389/fnut.2021.742081.
19. Abdala, R.; del Valle, E.E.; Negri, A.L.; Bridoux, P.; Paganti, L.G.; Bravo, M.; Sintado, L.; Di Rienzo, P.; Schiavelli, O.R.; Zanchetta, M.B.; et al. Sarcopenia in hemodialysis patients from Buenos Aires, Argentina. *Osteoporos. Sarcopenia* **2021**, *7*, 75–80. https://doi.org/10.1016/j.afos.2021.04.001.

20. Yuenyongchaiwat, K.; Jongritthiporn, S.; Somsamarn, K.; Sukkho, O.; Pairojkittrakul, S.; Traitanon, O. Depression and low physical activity are related to sarcopenia in hemodialysis: A single-center study. *PeerJ* **2021**, *9*, e11695. https://doi.org/10.7717/peerj.11695.
21. Cheng, D.; Zhang, Q.; Wang, Z.; Li, J.; Jian, G.; Wang, N. Association Between Sarcopenia and Its Components and Dependency in Activities of Daily Living in Patients on Hemodialysis. *J. Ren. Nutr.* **2021**, *31*, 397–402. https://doi.org/10.1053/j.jrn.2020.08.016.
22. Matsuzawa, R.; Yamamoto, S.; Suzuki, Y.; Imamura, K.; Harada, M.; Matsunaga, A.; Tamaki, A.; Fukui, T.; Shimokado, K. The clinical applicability of ultrasound technique for diagnosis of sarcopenia in hemodialysis patients. *Clin. Nutr.* **2020**, *40*, 1161–1167. https://doi.org/10.1016/j.clnu.2020.07.025.
23. Rosa, C.S.C.; Msc, H.S.R.; Vogt, B.P.; Sakkas, G.K.; Monteiro, H.L. Sarcopenia diagnosis in patients receiving hemodialysis: Agreement among different consensuses. *Nutr. Clin. Pr.* **2021**, Epub ahead of print: 30-12-2021. https://doi.org/10.1002/ncp.10813.
24. Lin, Y.-L.; Wang, C.-H.; Tsai, J.-P.; Chen, C.-T.; Chen, Y.-H.; Hung, S.-C.; Hsu, B.-G. A Comparison of SARC-F, Calf Circumference, and Their Combination for Sarcopenia Screening among Patients Undergoing Peritoneal Dialysis. *Nutrients* **2022**, *14*, 923. https://doi.org/10.3390/nu14050923.
25. Yasar, E.; Tek, N.A.; Tekbudak, M.Y.; Yurtdaş, G.; Gülbahar, Ö.; Uyar, G..; Ural, Z.; Çelik, M.; Erten, Y. The Relationship Between Myostatin, Inflammatory Markers, and Sarcopenia in Patients With Chronic Kidney Disease. *J. Ren. Nutr.* **2022**, S1051-2276(22)00011-5. https://doi.org/10.1053/j.jrn.2022.01.011.
26. Ding, Y.; Chang, L.; Zhang, H.; Wang, S. Predictive value of phase angle in sarcopenia in patients on maintenance hemodialysis. *Nutrition* **2021**, *94*, 111527. https://doi.org/10.1016/j.nut.2021.111527.
27. Kurajoh, M.; Mori, K.; Miyabe, M.; Matsufuji, S.; Kizu, A.; Tsujimoto, Y.; Emoto, M. Xanthine Oxidoreductase Inhibitor Use Associated With Reduced Risk of Sarcopenia and Severe Sarcopenia in Patients Undergoing Hemodialysis. *Front. Med.* **2022**, *9*, 817578. https://doi.org/10.3389/fmed.2022.817578.
28. Isoyama, N.; Qureshi, A.R.; Avesani, C.M.; Lindholm, B.; Bàràny, P.; Heimbürger, O.; Cederholm, T.; Stenvinkel, P.; Carrero, J.J. Comparative Associations of Muscle Mass and Muscle Strength with Mortality in Dialysis Patients. *Clin. J. Am. Soc. Nephrol.* **2014**, *9*, 1720–1728. https://doi.org/10.2215/cjn.10261013.
29. Kittiskulnam, P.; Chertow, G.M.; Carrero, J.J.; Delgado, C.; Kaysen, G.A.; Johansen, K.L. Sarcopenia and its individual criteria are associated, in part, with mortality among patients on hemodialysis. *Kidney Int.* **2017**, *92*, 238–247. https://doi.org/10.1016/j.kint.2017.01.024.
30. Kamijo, Y.; Kanda, E.; Ishibashi, Y.; Yoshida, M. Sarcopenia and Frailty in PD: Impact on Mortality, Malnutrition, and Inflammation. *Perit. Dial. Int.* **2018**, *38*, 447–454. https://doi.org/10.3747/pdi.2017.00271.
31. Giglio, J.; Kamimura, M.A.; Lamarca, F.; Rodrigues, J.; Santin, F.; Avesani, C.M. Association of Sarcopenia with Nutritional Parameters, Quality of Life, Hospitalization, and Mortality Rates of Elderly Patients on Hemodialysis. *J. Ren. Nutr.* **2018**, *28*, 197–207. https://doi.org/10.1053/j.jrn.2017.12.003.
32. Kim, J.-K.; Kim, S.G.; Oh, J.-E.; Lee, Y.-K.; Noh, J.-W.; Kim, H.J.; Song, Y.R. Impact of sarcopenia on long-term mortality and cardiovascular events in patients undergoing hemodialysis. *Korean J. Intern. Med.* **2019**, *34*, 599–607. https://doi.org/10.3904/kjim.2017.083.
33. Mori, K.; Nishide, K.; Okuno, S.; Shoji, T.; Emoto, M.; Tsuda, A.; Nakatani, S.; Imanishi, Y.; Ishimura, E.; Yamakawa, T.; et al. Impact of diabetes on sarcopenia and mortality in patients undergoing hemodialysis. *BMC Nephrol.* **2019**, *20*, 105. https://doi.org/10.1186/s12882-019-1271-8.
34. Lin, Y.-L.; Liou, H.-H.; Wang, C.-H.; Lai, Y.-H.; Kuo, C.-H.; Chen, S.-Y.; Hsu, B.-G. Impact of sarcopenia and its diagnostic criteria on hospitalization and mortality in chronic hemodialysis patients: A 3-year longitudinal study. *J. Formos. Med. Assoc.* **2019**, *119*, 1219–1229. https://doi.org/10.1016/j.jfma.2019.10.020.
35. Chiang, J.M.; Kaysen, G.A.; Segal, M.; Chertow, G.M.; Delgado, C.; Johansen, K.L. Low testosterone is associated with frailty, muscle wasting and physical dysfunction among men receiving hemodialysis: A longitudinal analysis. *Nephrol. Dial. Transplant.* **2018**, *34*, 802–810. https://doi.org/10.1093/ndt/gfy252.
36. Macedo, C.; Amaral, T.F.; Rodrigues, J.; Santin, F.; Avesani, C.M. Malnutrition and Sarcopenia Combined Increases the Risk for Mortality in Older Adults on Hemodialysis. *Front. Nutr.* **2021**, *8*, 721941. https://doi.org/10.3389/fnut.2021.721941.
37. Souweine, J.S.; Pasquier, G.; Kuster, N.; Rodriguez, A.; Patrier, L.; Morena, M.; Badia, E.; Raynaud, F.; Chalabi, L.; Raynal, N.; et al. Dynapaenia and sarcopaenia in chronic haemodialysis patients: Do muscle weakness and atrophy similarly influence poor outcome? *Nephrol. Dial. Transpl.* **2021**, *36*, 1908–1918.
38. Kim, C.; Kim, J.-K.; Lee, H.-S.; Kim, S.G.; Song, Y.R. Longitudinal changes in body composition are associated with all-cause mortality in patients on peritoneal dialysis. *Clin. Nutr.* **2020**, *40*, 120–126. https://doi.org/10.1016/j.clnu.2020.04.034.
39. Davenport, A. Comparison of frailty, sarcopenia and protein energy wasting in a contemporary peritoneal dialysis cohort. *Perit. Dial. Int.* **2022**, 8968608221077462. https://doi.org/10.1177/08968608221077462.
40. Ferreira, M.F.; Böhlke, M.; Pauletto, M.B.; Frühauf, I.R.; Gonzalez, M.C. Sarcopenia diagnosis using different criteria as a predictor of early mortality in patients undergoing hemodialysis. *Nutrition* **2022**, *95*, 111542. https://doi.org/10.1016/j.nut.2021.111542.

41. Ishimura, E.; Okuno, S.; Nakatani, S.; Mori, K.; Miyawaki, J.; Okazaki, H.; Sugie, N.; Norimine, K.; Yamakawa, K.; Tsujimoto, Y.; et al. Significant Association of Diabetes With Mortality of Chronic Hemodialysis Patients, Independent of the Presence of Obesity, Sarcopenia, and Sarcopenic Obesity. *J. Ren. Nutr.* **2022**, *32*, 94–101. https://doi.org/10.1053/j.jrn.2021.07.003.
42. Sánchez-Tocino, M.L.; Miranda-Serrano, B.; Gracia-Iguacel, C.; De-Alba-Peñaranda, A.M.; Mas-Fontao, S.; López-González, A.; Villoria-González, S.; Pereira-García, M.; Ortíz, A.; González-Parra, E. Sarcopenia assessed by 4-step EWGSOP2 in elderly hemodialysis patients: Feasibility and limitations. *PLoS ONE* **2022**, 17, e0261459. https://doi.org/10.1371/journal.pone.0261459.
43. Hayashi, H.; Izumiya, Y.; Hayashi, O.; Ichii, M.; Tsujimoto, Y.; Yoshiyama, M. Dynapenia is an independent predictor of cardio-cerebrovascular events in patients undergoing hemodialysis. *Hear Vessel.* **2022**, *37*, 1066–1074. https://doi.org/10.1007/s00380-021-02006-7.
